# Sex Matters: Effects of Sex and Mating in the Presence and Absence of a Protective Microbe

**DOI:** 10.3389/fcimb.2021.713387

**Published:** 2021-10-07

**Authors:** Anke Kloock, Lena Peters, Charlotte Rafaluk-Mohr

**Affiliations:** Department of Zoology, University of Oxford, Oxford, United Kingdom

**Keywords:** defensive mutualism, sexual immune dimorphism, microbe-mediated protection, host–pathogen interaction, heterogeneity, protection

## Abstract

In most animals, female investment in offspring production is greater than for males. Lifetime reproductive success (LRS) is predicted to be optimized in females through extended lifespans to maximize reproductive events by increased investment in immunity. Males, however, maximize lifetime reproductive success by obtaining as many matings as possible. In populations consisting of mainly hermaphrodites, optimization of reproductive success may be primarily influenced by gamete and resource availability. Microbe-mediated protection (MMP) is known to affect both immunity and reproduction, but whether sex influences the response to MMP remains to be explored. Here, we investigated the sex-specific differences in survival, behavior, and timing of offspring production between feminized hermaphrodite (female) and male *Caenorhabditis elegans* following pathogenic infection with *Staphylococcus aureus* with or without MMP by *Enterococcus faecalis*. Overall, female survival decreased with increased mating. With MMP, females increased investment into offspring production, while males displayed higher behavioral activity. MMP was furthermore able to dampen costs that females experience due to mating with males. These results demonstrate that strategies employed under pathogen infection with and without MMP are sex dependent.

## Introduction

In dioecious organisms, females and males invest energy differently throughout their life: In most sexually reproducing species, females and males pay different costs for mating (Rolff, 2002; Rolff et al., 2005). While males invest more energy into mating [by displaying mate searching behavior (Lipton et al., 2004) or outcompeting other males and their sperm (LaMunyon and Ward, 1995)], females generally invest more energy into producing high-quality offspring (Bateman, 1948; Trivers, 1972; Clutton-Brock, 1988). Females try to maximize their reproductive lifespan, while males maximize their sperm output, which is described as Bateman’s principle (Bateman, 1948; Trivers, 1972; Clutton-Brock, 1988). Due to these differences in energy investment into mating, the costs and benefits of investment into immunity differ between sexes (Sheldon and Verhulst, 1996; McKean and Nunney, 2001; Lindsey and Altizer, 2009). While males of different species have a reduced survival and body size upon infection with pathogens (such as *Panorpa vulgaris*, *Caenorhabditis elegans*, and *Daphnia magna* after infection with *Micrococcus luteus*, *Bacillus thuringiensis*, and *Octosporea bayeri*, respectively), females tend to invest more heavily in immunity (Kurtz et al., 2000; Roth et al., 2008; Zuk, 2009; Masri et al., 2013). Female scorpion flies have increased immune activity (Kurtz et al., 2000), and women survive pandemics (Zuk, 2009), slavery, or famines better than men do (Zarulli et al., 2018). Males, on the other hand, tend to invest more heavily in behavioral traits such as mate-search behavior (Hemptinne et al., 1996; Lipton et al., 2004) and pathogen avoidance (Masri et al., 2013).

In species where the majority of individuals are self-fertilizing hermaphrodites, the situation is more complex as mating is not necessary to maximize fecundity (Brenner, 1974), although similarly to females (Bateman, 1948), fecundity is likely to be primarily dependent on energy availability if gametes are not limited. In self-fertilizing hermaphrodites, such as *C. elegans*, for whom mating is not necessary for reproduction but males remain in the population, hermaphrodites may be even more “coy” and “choosy” than females in dioecious populations, potentially causing more extreme differences in investment strategies following pathogen infection.

We thus hypothesized that the benefit of a protective microbe would differ among sexes. Protective microbes can be important in host defense in the face of infection, a phenomenon referred to as “defensive mutualism” (King, 2019), where microbes can supplement the host’s immune system (Abt and Artis, 2013). Defensive mutualisms have been observed across kingdoms [reviewed in (Ford and King, 2016)]. The potential of defensive mutualism to enhance survival as well as offspring production has been observed repeatedly (Koehler et al., 2013; King et al., 2016; Kloock et al., 2020). However, most of these examples have only considered population-level effects, while few studies have focused on individual behaviors and/or sex differences between the hosts (McLean et al., 2018).

To test for differences in survival, behavior, and timing of offspring production between the two host sexes with or without microbe-mediated protection (MMP) during infection, we used an established experimental system using *Caenorhabditis elegans* as a host, *Enterococcus faecalis* as a protective microbe, and pathogenic *Staphylococcus aureus* (Ford et al., 2016; King et al., 2016; Rafaluk-Mohr et al., 2018). Once *C. elegans* worms are exposed to *E. faecalis* and *S. aureus*, *E. faecalis* provides microbe-mediated protection (MMP) by scavenging siderophores from *S. aureus* (Ford et al., 2016). Here, we used a population of *C. elegans* made up of males and feminized hermaphrodites, which only carry eggs and cannot produce sperm, and thus are referred to as females (Theologidis et al., 2014). To test for the effect of mating on traits such as survival after pathogen infection and over a lifetime, behavior upon different mating and population structures, and the timing of offspring production, we separated females and males and manipulated the time frame that they could come into contact and mate to be either unmated, short-term mated, or lifetime mated. To test the impact of MMP, worms were exposed to one of three bacterial diets: food only, pathogen without MMP, or pathogen with MMP. We investigated the differences in survival, behavior, and timing of offspring production for the two sexes under the different mating and bacterial diet conditions. We predicted that feminized hermaphrodites would benefit more from the provision of MMP, as feminized hermaphrodites experience costs of the provision of eggs and mating. We furthermore predicted that males would benefit differently from the provision of MMP either by differences in survival or mate searching activity. Our predictions were met, when we observed, that females highly benefit from MMP and show increased survival after pathogen infection and differences in offspring timing, while males are benefiting from MMP by displaying higher mate searching behavior. MMP is able to dampen the costs that females pay by being mated by males.

## Materials and Methods

### Worm and Bacteria System

We used an obligate outcrossing worm population [line EEVD00 from Henrique Teotonio (Theologidis et al., 2014)] where worms carry the *fog-2(q71)* mutation, preventing hermaphrodites from producing sperm (Theologidis et al., 2014). Worms were kept on Nematode Growth Medium (NGM) (Brenner, 1974), and fed with nonpathogenic *Salmonella enterica*, hereafter referred to as food (Diaz et al., 2015; Desai et al., 2019; Kloock et al., 2020). For pathogenic infection, the Gram-positive *Staphylococcus aureus* strain MSSA476 (Holden et al., 2004) was used. The strain OG1RF of *Enterococcus faecalis* (Garsin et al., 2001) was used as a protective microbe against *S. aureus* infection (Ford et al., 2016; King et al., 2016; Rafaluk-Mohr et al., 2018). *E. faecalis* positively affects host survival, even in the absence of the pathogen *S. aureus* (Kloock et al., 2020).

### Pathogenic Infection and Long-Term Survival Analysis

All assays were carried out blind. All results shown for food and pathogenic infection with or without MMP have been generated from different setups for each bacterial diet. Results thus cannot be compared between different diets but only within.

Worms were sterilized and synchronized *via* bleaching (Stiernagle, 2006). Simultaneously, the bacteria were grown in overnight cultures: either *E. faecalis* overnight in 25 ml of Todd-Hewitt Broth (THB) or food in 25 ml of Lysogeny broth (LB), both at 30°C in a shaking incubator. NGM plates (6 cm) were inoculated with either 400 µl of food or 200 µl of food mixed with 200 µl of *E. faecalis*. A total of 600 L1 worms were added to each NGM plate and kept at 20°C for 42 h. Simultaneously, a liquid culture of *S. aureus* was grown in THB from a frozen stock, while food was grown in LB. Both cultures were incubated under shaking conditions at 30°C overnight. The following day, 20 µl of *S. aureus* overnight culture was pipetted onto 3 cm on Tryptone Soy Broth agar (TSB) plates and incubated at 30°C overnight. Simultaneously, 6cm NGM plates were inoculated with 150µl food. These plates were used to split worms into groups of only females, only males, or 50:50 mixed for 6–8 h (time point when the first eggs appeared on the plate) (**Table S1**). After the worms had mated, 50 worms were placed onto the *S. aureus* lawn with a platinum wire pick and left at 25°C for 24 h (**Figure S1**) in groups of unmated, short-term mated, and lifetime mated individuals of both sexes (**Table S1**).

Survival upon pathogenic infection was scored after 24 h. Worms were considered dead if they did not respond to touch with a platinum wire pick. After survival was scored, 10 worms were transferred to 3cm NGM plates seeded with 150µl food and placed at 25°C. Worms were transferred to new plates every 24 h with a platinum wire until no further offspring production occurred. Survival was scored every day until all worms were dead. For the food-alone treatment, the long-term survival assay followed a similar protocol except that the experimental procedure was carried out at 20°C, as is standard for *C. elegans* (Amrit et al., 2014).

### Activity Analysis

Males and females were previously observed to show different activities, may it be due to infection (Masri et al., 2013) or due to higher mate searching behavior (Lipton et al., 2004). After 24 h on the pathogen infection plates, worm behavioral activity was determined *via* calculating the fraction of worms at the edge of the plate. Worms were considered at the edge of the plate if they could not be seen from above.

### Avoidance Analysis

Avoidance behavior is known as a response to pathogen defense (Pees et al., 2017). The proportion of missing worms was calculated 24 h after pathogen infection and at each transfer in the long-term survival analysis, as previously described (Pees et al., 2017). To define the proportion of missing worms, the number of initially exposed worms minus the counted alive and counted dead worms was divided by the number of initially exposed worms. For the long-term avoidance analysis, the cumulative number of dead worms was used, while only the last time point of each experiment was plotted (**Figure S2**).

### Offspring Production

The presence or absence of viable offspring on a plate was noted during pathogen infection and during long-term survival. Unmated females might sometimes produce and lay unfertilized eggs, which do not develop into viable larvae (Singaravelu and Singson, 2011). Due to feasibility, the exact numbers of viable offspring could not be assessed. Offspring production was defined as a proportion of plates that had viable offspring over the total amount of plates per treatment.

### Statistical Analysis

Statistical analyses were carried out with RStudio (Version 1.1.463 for Mac) (RStudio Team, 2020). Figures were created with the ggplot2 package (Version 2.1.0). All data, except for the long-term survival and offspring production data, were analyzed with generalized linear models to test for an effect of sex, the mating status, or an interaction between the two. If the interaction had a significant effect, a Tukey multiple-comparison tests (R package multcomp) (Hothorn et al., 2008) was performed (**Table S1**). The long-term survival data were analyzed with Kaplan–Meier log rank test with FDR correction for multiple testing (Therneau and Grambsch, 2002; Therneau, 2020). The offspring data were analyzed using a Wilcoxon rank test (Mann and Whitney, 1947).

## Results and Discussion

### Lifetime Mated Females Are Most Affected by Pathogen Infection and Mating

**Figure 1:**
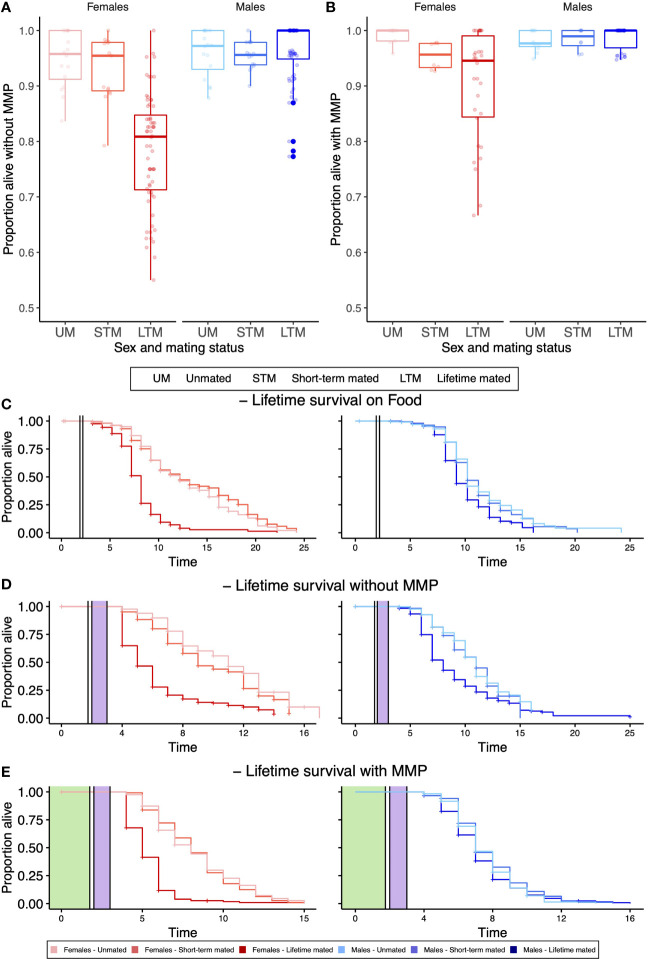
Survival of different sexes and mating treatments after 24-h pathogen infection **(A, B)** and lifelong **(C–E)**. **(A)** Without MMP, females suffer more from mating than males do, while males survive better overall. **(B)** With MMP, females are suffering more from mating, while males survive better overall. **(C)** When only ever being exposed to food (in white), lifetime mated females survive worse than any other females, while only lifetime mated males survive worse than short-term mated males. **(D)** After pathogen infection (in purple) without MMP, lifetime mated females survive worse than short-term mated females, which survive worse than unmated females. Male survival was not affected by mating, while males live overall longer than females do. **(E)** After pathogen infection with MMP (in green), lifetime mated females survive worse than short-term mated females, while males are not affected by mating. **(A, B)** Boxplots display four biological replicates and three or four technical replicates with 50 worms on each plate. **(C–E)** Each curve represents the Kaplan–Meier survival estimate for three or four technical replicates and four or five biological replicates with 20 **(C)** or 10 worms **(D, E)** on each plate. Vertical lines indicate when worms were transferred to a new bacterial diet.

During pathogen infection without (**Figure 1A**) and with MMP (**Figure 1B**), males survived better than females (GLM, X^2^ = 172.383, df=1, p<0.001, and GLM, X^2^ = 34.383, df=1, p<0.001, respectively), while over a lifetime, this pattern was only present without MMP (**Figures 1C–E**; without MMP: p<0.001, Kaplan–Meier survival estimates (KMSE); on food: p=0.62 (KMSE), with MMP: p=0.32, (KMSE)). Females were dramatically affected by the mating status, during pathogen infection without and with MMP (p<0.001, GLM, X^2^ = 108.757, df=2, p<0.001 (**Figure 1A**), GLM, X^2^ = 27.152, df=2, p<0.001 (**Figure 1B**), respectively) and also over a lifetime independent of the bacterial diet (**Figures 1C–E**), where lifetime mated females survived worse than their unmated or short-term mated counterparts (on food, without MMP and with MMP both comparisons p<0.001). Males were not affected by mating neither during pathogen infection nor over a lifetime (all p>0.05).

Females suffer worse the longer they have been mated with males, while males survive pathogen infection better than females independent of MMP (**Figures 1A, B**). This pattern holds during pathogen infection (**Figures 1A, B**) and over a lifetime (**Figures 1C–E**). The extreme differences we see here may be partially explained by the fact that the females used in our experiments evolved at least in their recent evolutionary history as hermaphrodites that would only occasionally outcross with males and thus are adapted to a far lower frequency of mating (Theologidis et al., 2014). The potential of MMP to enhance survival (King et al., 2016; Martinez et al., 2016; Kloock et al., 2020) as well as offspring production (Koehler et al., 2013) has been shown repeatedly. We expand on this work by investigating whether MMP can mitigate the costs that individuals pay for mating with males. So far, these effects have mainly been considered at the population level. However, the role of MMP might have different effects on individual behaviors of different sexes in different life stages (McLean et al., 2018).

A potential explanation for the observed phenotype could be mechanical gut integrity, which can be different between males and females as observed in *Drosophila* (Regan et al., 2016). The pathogen used here, *S. aureus*, is known to accumulate in the worms’ gut and to kill worms by distention of the intestinal lumen (Sifri et al., 2003). If gut integrity would thus be more easily damaged in *C. elegans* females, but not in males (personal observation, **Figure S3**), this could serve as a potential explanation as to why females are more harshly affected by pathogenic infection with *S. aureus*. The potentially disrupted gut integrity by *S. aureus* infection could further be weakened by mechanical penetration by males, which can shorten hermaphrodites’ lifespan (Gems and Riddle, 1996). Whether this hypothesis holds true remains to be tested. Although it is possible that due to different gut structures the absolute numbers of protective and pathogenic microbes might differ, we do know that both sexes benefit from protection and due to proliferation in the gut and colonization levels in both sexes are high (**Figure S5**). Although females carry significantly higher protective microbe loads than males (LM, Sum Sq = 24.552, Df=1, F-value=17.6003, p<0.001), females are 30% larger than males (Riddle et al., 1997), which is proportional to the differences seen in bacterial loads (**Figure S5**). We thus would expect levels of protection to be similar.

In the absence of hermaphrodites, *C. elegans* males show highly reduced lifespan (Gems and Riddle, 1996; Shi et al., 2017). The presence of only the male pheromones was sufficient to shorten hermaphrodite (Maures et al., 2014) and male lifespan (Shi et al., 2017). We can, however, only observe this effect for the food-alone treatment (**Figure 1C**) when lifetime mated males survive significantly longer than unmated or short-term mated males (**Figure 1C** and **Table S2**). This life-shortening effect of single-housed males could not be observed after pathogen infection with or without MMP (**Figures 1D, E**). Both the infection with *S. aureus* and mating reduce the amount of lipids in the worm (Shi et al., 2017; Dasgupta et al., 2020). Possibly the lack of male lifespan reduction in single sex plates after pathogen infection could be linked to this lipid reduction, even though further studies are needed to determine this effect.

The act of mating is costly and life shorting (Gems and Riddle, 1996), independent of offspring production, as here short-term mated females that also produce costly offspring do not have lower survival than unmated females over a lifetime (**Figures 1C–E**) (Maures et al., 2014; Shi and Murphy, 2014). In *C. elegans*, the life-shortening effect is not solely due to offspring production, as even without physical contact or successful mating, hermaphrodites showed reduced lifespan (Maures et al., 2014). During mating, males transfer seminal fluids alongside sperm, which reduces female life in *C. elegans* and other species and can leave females immune-suppressed post mating (Rolff and Siva-Jothy, 2003). Furthermore, the presence of MMP is able to dampen the costs that females have to pay by being mated with males. Our results reflect a wealth of findings in other species that has shown mating to be costly, such as in *Drosophila* (Fowler and Partridge, 1989) or birds (Liker and Székely, 2005).

### Male and Female Behavior Is Linked to the Presence of the Other Sex

**Figure 2:**
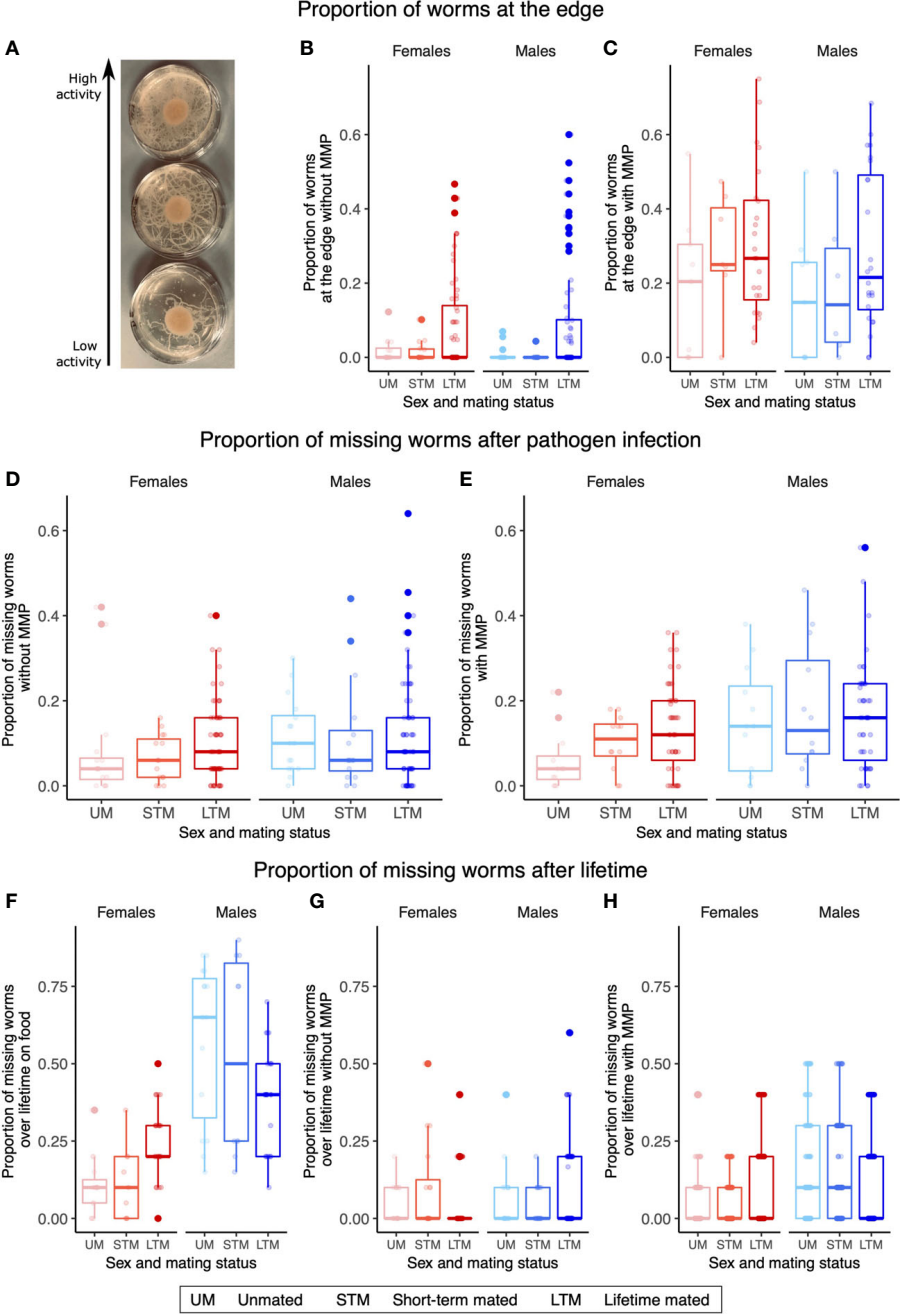
Female and male behavior is linked to the presence of the other sex. **(A)** Picture of three pathogenic plates with different activity levels from low to high. **(B)** Without MMP after pathogen infection, plates with lifetime mated worms of both sexes had a higher proportion of worms at the edge of the plate. **(C)** With MMP after pathogen infection, plates with lifetime mated worms of both sexes have a higher proportion of worms at the edge of the plate. Proportion of missing worms of different sexes and mating treatments either during pathogen infection **(D, E)** and over lifetime (**F–H**, only the last time point is plotted). **(D)** During pathogen infection without MMP, a higher proportion of males are missing than of females. **(E)** During pathogen infection with MMP, more males went missing than females. **(F)** Proportion of missing worms on food over lifetime. Females showed lower proportion of missing worms than males. **(G)** Proportion of missing worms without MMP over lifetime with no differences detected. **(H)** Proportion of missing worms with MMP over lifetime, where males have a higher proportion of missing worms than females do. **(B–H)** Boxplots represent the mean ± the standard error of the mean of four or five biological replicates and three or four technical replicates with 50 **(B–E)**, 20 **(F)**, or 10 worms **(G, H)** on each plate. **(F–H)** Changes in the proportion of missing worms over time can be found in **Figure S2**.

Females and males respond differently to infection and also display different behavior in the face of infection. Without MMP, those plates with both sexes display higher activity than those plates with unmated or short-term mated individuals (GLM, X^2^ = 197.14, df=5, p<0.001, **Figure 2B**), while with MMP, unmated individuals showed significantly decreased activity in comparison to unmated or short-term mated individuals (GLM, X^2^ = 47.812.14, df=2, p<0.001, **Figure 2C**). As this increased activity for lifetime mating worms can be a hint to increased avoidance behavior, which is itself a mechanism to respond to pathogenic infection (Pees et al., 2017), we also assessed the proportion of missing worms during pathogen infection (**Figures 2D, E**) and over a lifetime (**Figures 2F–H**). Males went missing with a higher proportion than females during pathogen infection independent of MMP (without MMP: GLM, X^2^ = 6.4367, df=1, p=0.04002 and with MMP: GLM, X^2^ = 37.903, df=1, p<0.001) and over a lifetime on food alone (GLM, X^2^ = 195.959, df=1, p<0.001) and with MMP (GLM, X^2^ = 160.466, df=1, p=0.002), while the difference between the sexes was not significant after pathogen infection without MMP (GLM, X^2^ = 2.6875, df=1, p=0.10).

The activity level was mainly determined by the mating status, while the proportion of missing worms was predominantly determined by sex (**Figure 2**). Infected females might not be able to move around as much as healthy females, as only healthy females and males on food alone show effects of mating (**Figure 2**). Furthermore, due to their evolutionary history as hermaphrodites, females may have a decreased drive to look for mates, as mate searching would be largely unnecessary in wild-type populations of self-fertilizing hermaphrodites. The mating status also affected the proportion of missing worms in opposite directions in lifetime mated worms on food: females would be missing with a higher proportion with males present while males would stay with a higher proportion with females present. A parsimonious explanation is that males exhibit higher mate searching behavior, and this is increased when females are not present (Lipton et al., 2004). The proportion of missing worms observed here does not appear to be a consequence of pathogen avoidance behavior (Pees et al., 2017), as this behavior is not observed for pathogen infection without MMP but might instead be dependent on the population structure on the plate.

### MMP Enables Females to Invest in Offspring Production During Pathogenic Infection

**Figure 3:**
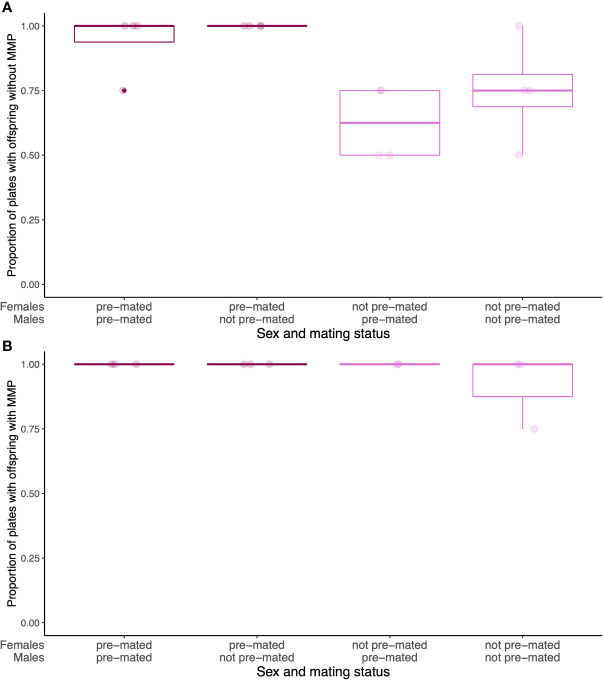
Differences in timing of offspring production during pathogen infection without **(A)** and with **(B)** MMP **(A)**. During pathogen infection but without MMP, there are fewer plates with offspring, despite the presence of males. **(B)** During pathogen infection, but with MMP, all plates with pre-mated and not pre-mated females are producing offspring. Boxplots represent three or four technical replicates with 25 females and 25 males on each plate.

Pre-mated females and non-pre-mated females did not start to produce offspring at the same time (**Figure 3**). Without MMP, pre-mated females did not start to produce offspring during pathogen infection, while pre-mated females did (Wilcoxon rank test (WRT), W=57.5, p=0.004, **Figure 3A**). In the presence of MMP, all females start producing offspring at the same time (WRT, W=18, p=1, **Figure 3B**), independent of their pre-mating status. This pattern could indicate that if females have not yet been mated prior to pathogen infection without MMP, they do not pursue offspring production during infection. They might potentially put reproduction on hold during less favorable conditions and resume reproduction once conditions are more favorable. This can be observed, when over a lifetime, pre-mated and not pre-mated females do not differ in the length of offspring production on each bacterial diet (**Figure S4**). This is in contrast to previous findings that show fecundity compensation in response to *S. aureus* infection in *C. elegans* (Pike et al., 2019). This is likely because we only scored for the presence/absence of offspring at a set time point and thus would not be able to detect any change of timing or amount in offspring production, as was described for fecundity compensation in this system of *C. elegans* and *S. aureus* (Pike et al., 2019).

## Conclusion

In conclusion, female survival decreases with increasing mating with males, while male survival was unaffected by mating after pathogen infection. The two sexes benefit from MMP differently. With MMP, females invest more energy into egg production, while males invest more into mate searching behavior. These results are likely to be enhanced in the results we see here as females were artificially feminized, having evolved as hermaphrodites. Furthermore, MMP is able to dampen the costs that females pay by being mated by males. This study highlights that in spite of consistent population level responses to defensive mutualists, individual variation depends heavily on diet, sex, mating status, and interaction of these factors. Even though defensive mutualists provide benefits to the host, for females, mating comes at a high cost.

## Data Availability Statement

The datasets presented in this study can be found in online repositories. The names of the repository/repositories and accession number(s) can be found below: https://osf.io/bnwrt/, Open Science Framework.

## Author Contributions

AK and CR-M conceived and designed the project. AK and LP performed all experiments. AK and CR-M performed the statistical analysis. AK and CR-M wrote the manuscript and all authors agreed on the final version. All authors contributed to the article and approved the submitted version.

## Funding

AK was supported by a fellowship from the “Studienstiftung des Deutschen Volkes”. LP was supported by an ERASMUS + fellowship. This work was funded by a Leverhulme Trust project grant (RPG‐2015‐165) to Kayla King.

## Conflict of Interest

The authors declare that the research was conducted in the absence of any commercial or financial relationships that could be construed as a potential conflict of interest.

## Publisher’s Note

All claims expressed in this article are solely those of the authors and do not necessarily represent those of their affiliated organizations, or those of the publisher, the editors and the reviewers. Any product that may be evaluated in this article, or claim that may be made by its manufacturer, is not guaranteed or endorsed by the publisher.

## Supplementary Material

Supplementary Figure 1 Experimental procedure. Starved worms were transferred to new NGM plates seeded with food 2.5 days before bleaching to allow for egg laying. Worms were then bleached as described previously (Stiernagle, 2006) and left in M9 buffer overnight to allow for larvae to hatch. Simultaneously, the bacteria on which worms were raised to L4 stage were grown in overnight cultures: Either *E. faecalis* overnight in 25ml of Todd-Hewitt Broth (THB), or food in 25ml of Lysogeny broth (LB), both at 30 °C in a shaking incubator. Subsequently, 6cm plates with NGM medium were inoculated with either 400µl of food or 200µl of food mixed with 200µl of *E. faecalis*. Plates with freshly inoculated bacteria were dried at room temperature before approximately 600 L1 worms were added to each NGM plate and transferred to 20°C for 42h. At the same time, a liquid culture of *S. aureus* was grown in THB from frozen stock, while food was grown in LB. Both cultures were incubated under shaking conditions at 30°C overnight. The following day, 20µl of the *S. aureus* overnight culture was pipetted onto 3cm on Tryptone Soy Broth agar (TSB) plates. Simultaneously, 6cm NGM plates were inoculated with 150µl food. These plates were used to split worms into groups of only females, only males or 50:50 mixed for 6-8h (time point when the first eggs appeared on the plate) as outlined in **Table S1**. For *S. aureus* infection, 50 worms were picked on pathogen exposure plates and left at 25°C for 24h. Survival was scored after 24h with all alive and dead worms present on the plates counted. Worms were considered dead if they do not respond to touch with a platinum wire pick. After survival was scored, 10 worms were transferred to 3cm NGM plates seeded with 150µl food and placed at 25°C. Worms were then picked to new plates every 24h until no offspring was produced anymore, to allow for more accurate tracking of individuals. Survival was scored every day until all worms were dead. The lifelong survival assay on food alone was performed as described above, with a few adjustments. The experimental procedure was carried out at 20 °C, and worms were split only females, only males or 50:50 mixed when worms were 46h old. The mixed treatment only consisted of “Females only with Males only” and “50:50 Females with 50:50 Males”. Once eggs occurred on the plates, worms were transferred to 6cm NGM plates inoculated with 150µl of food.

Supplementary Figure 2 Livelong proportion of missing worms on food **(A)**, with microbe-mediated protection **(B)** and without of microbe-mediated protection **(C)**. **(A)** Females showed lower proportion of missing worms than males on food alone (in white). **(B)** Proportion of missing worms after pathogen infection (in purple) without MMP over lifetime with no differences detected. **(C)** Proportion of missing worms with MMP (in green) over lifetime, where males have a higher proportion of missing worms than females do. **(A–C)** Each point represents the mean ± the standard error of the mean of four or five biological replicates and three or four technical replicates with 20(A) or 10 worms **(B, C)** on each plate.

Supplementary Figure 3 Females show hints of lost gut integrity, while male gut integrity is still intact. Worms that were fed food, coloured with a blue food dye (following a previously described protocol (Gelino et al., 2016). If gut integrity is still fully intact, blue dye can only be seen in the intestine (as in the lower worm – a male), while if the gut integrity is out of balance, the blue dye can be found in the whole worm body cavity (as in the upper worm – a female).

Supplementary Figure 4 Over a lifetime no difference between pre-mated and not pre-mated females for the days of offspring production can be observed independent on whether worms were raised on food (in orange), infected with the pathogen in the absence (in purple) or presence (in green) of MMP. **(A, B)** Each point represents the mean ± the standard error of the mean of three or four technical replicates.

Supplementary Figure 5 With MMP, females are colonized by more *E. faecalis* than their male counter parts. After 24h infection with *S. aureus*, worms were filter tip washed as described before (Kloock et al., 2020) to remove all externally attached bacteria. Consequently, worms were crushed open, and the gut content was plated on *E. faecalis* selective medium (TSA + rifampicin), to only count *E. faecalis* colonies. The difference between the two sexes is significant with a linear model (LM) (LM, Sum Sq = 24.552, Df = 1, F-value = 17.6003, p = 4.99x10^-5^), while neither the mating status nor an interaction of the two show significant differences (both p>0.05).

Supplementary Table 1 Mating Treatments: Three different mating treatments (Unmated, Short-term mated and Lifetime mated) were set up for both sexes (Female in Red and Male in Blue), which were either single sex plates for each sex (Females only or Males only) or a 50:50 mixed population. Worms were left on these mating plates for 6-8h, before three different mating treatments were set up (unmated, short-term mated, and lifetime mated, the darker the colour the longer the mating period) for each sex.

Supplementary Table 2 Summary of all statistical results. The following abbreviations were used: U, unmated; STM, short-term mated; LTM, Lifetime mated.
