# Supplementary figures and images for: Sex Matters: Effects of Sex and Mating in the Presence and Absence of a Protective Microbe
Source: Front Cell Infect Microbiol. 2021 Oct 7;11:713387. doi: 10.3389/fcimb.2021.713387 (PMC8529166; doi:10.3389/fcimb.2021.713387)

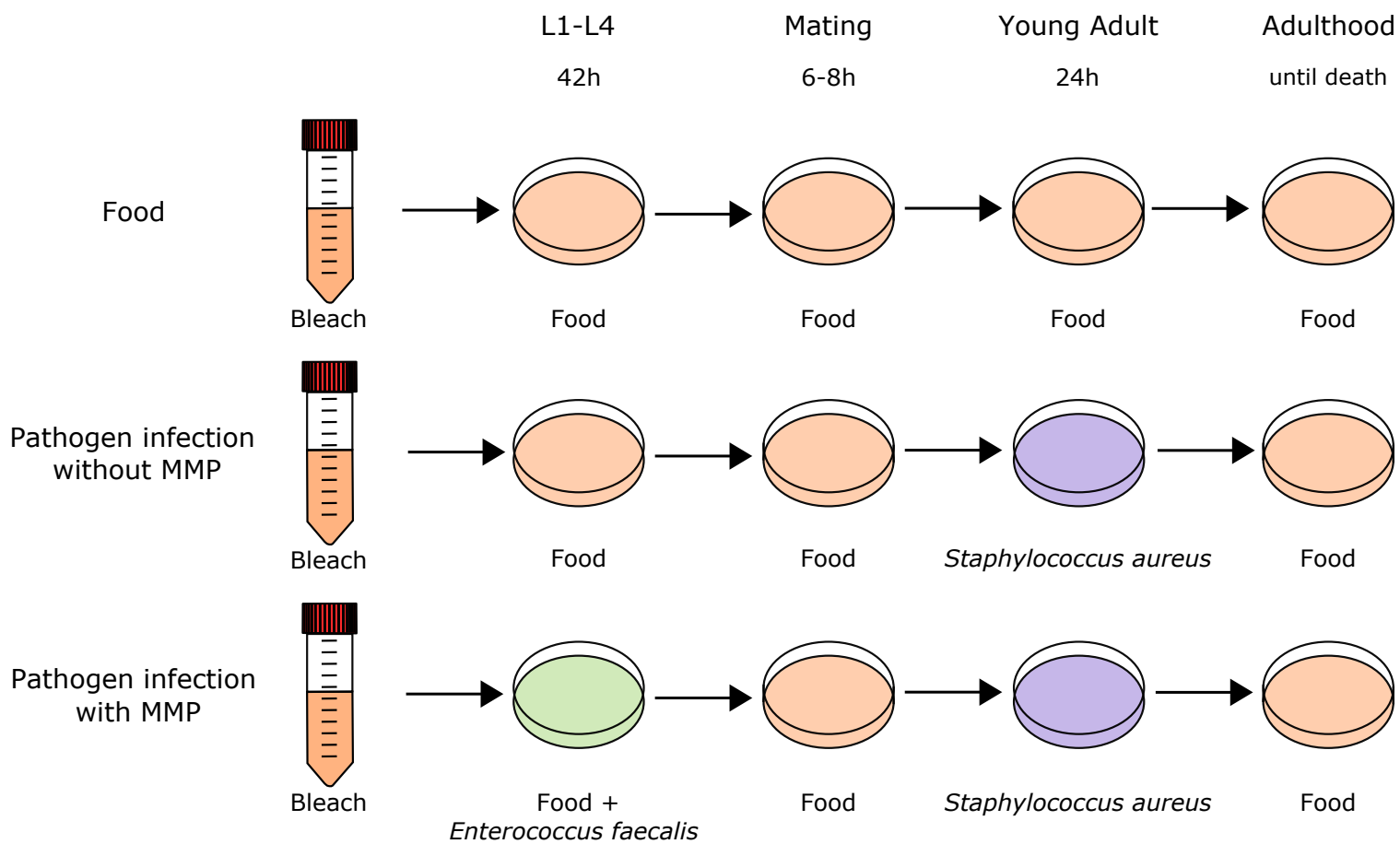

Supplement: Supplementary Figure 1 — Experimental procedure. Starved worms were transferred to new NGM plates seeded with food 2.5 days before bleaching to allow for egg laying. Worms were then bleached as described previously (Stiernagle, 2006) and left in M9 buffer overnight to allow for larvae to hatch. Simultaneously, the bacteria on which worms were raised to L4 stage were grown in overnight cultures: Either E. faecalis overnight in 25ml of Todd-Hewitt Broth (THB), or food in 25ml of Lysogeny broth (LB), both at 30 °C in a shaking incubator. Subsequently, 6cm plates with NGM medium were inoculated with either 400µl of food or 200µl of food mixed with 200µl of E. faecalis. Plates with freshly inoculated bacteria were dried at room temperature before approximately 600 L1 worms were added to each NGM plate and transferred to 20°C for 42h. At the same time, a liquid culture of S. aureus was grown in THB from frozen stock, while food was grown in LB. Both cultures were incubated under shaking conditions at 30°C overnight. The following day, 20µl of the S. aureus overnight culture was pipetted onto 3cm on Tryptone Soy Broth agar (TSB) plates. Simultaneously, 6cm NGM plates were inoculated with 150µl food. These plates were used to split worms into groups of only females, only males or 50:50 mixed for 6-8h (time point when the first eggs appeared on the plate) as outlined in Table S1 . For S. aureus infection, 50 worms were picked on pathogen exposure plates and left at 25°C for 24h. Survival was scored after 24h with all alive and dead worms present on the plates counted. Worms were considered dead if they do not respond to touch with a platinum wire pick. After survival was scored, 10 worms were transferred to 3cm NGM plates seeded with 150µl food and placed at 25°C. Worms were then picked to new plates every 24h until no offspring was produced anymore, to allow for more accurate tracking of individuals. Survival was scored every day until all worms were dead. The lifelong survival assay on food alone [file Image_1.pdf]

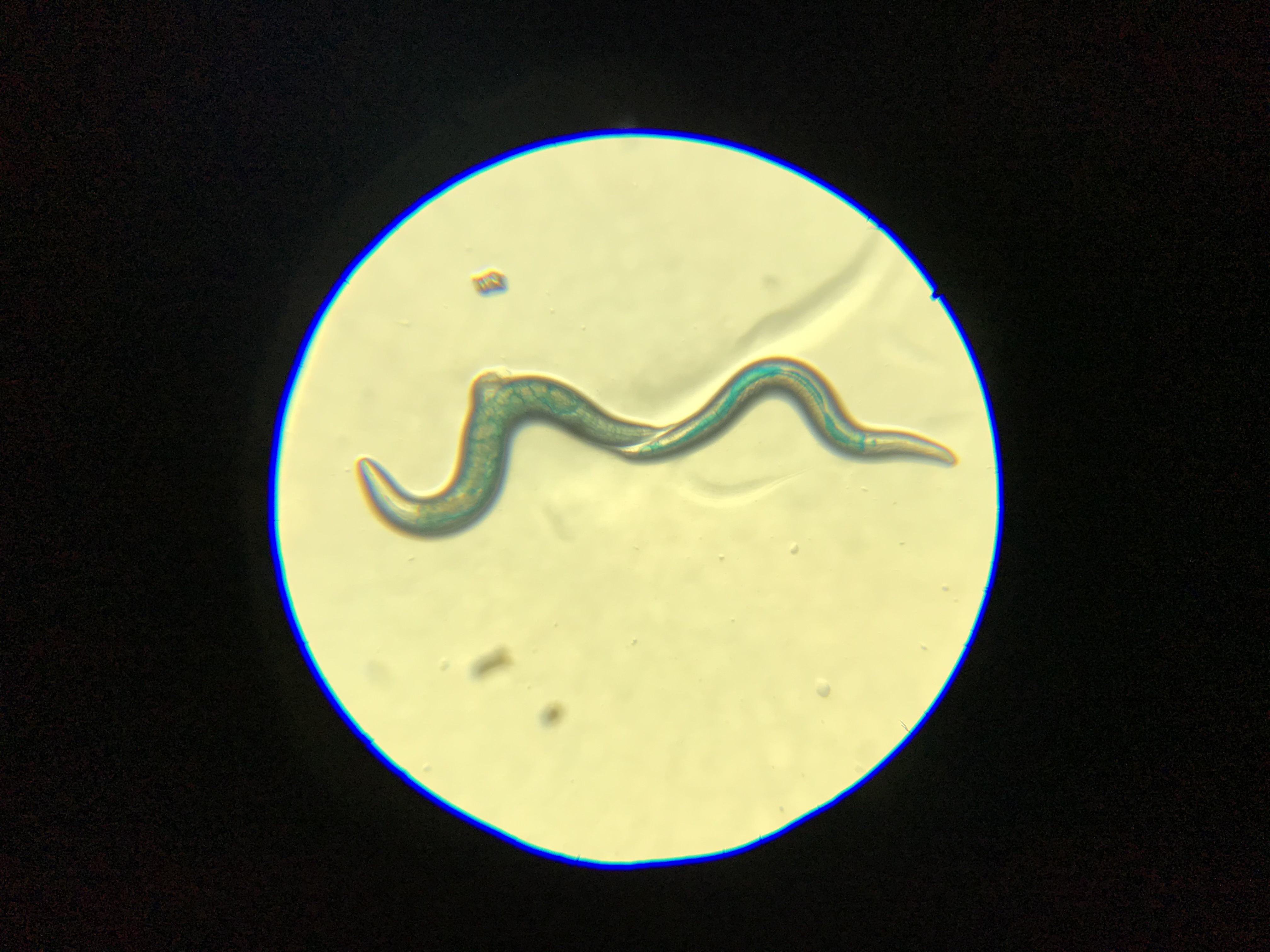

Supplement: Supplementary Figure 3 — Females show hints of lost gut integrity, while male gut integrity is still intact. Worms that were fed food, coloured with a blue food dye (following a previously described protocol (Gelino et al., 2016). If gut integrity is still fully intact, blue dye can only be seen in the intestine (as in the lower worm – a male), while if the gut integrity is out of balance, the blue dye can be found in the whole worm body cavity (as in the upper worm – a female). [file Image_3.jpeg]

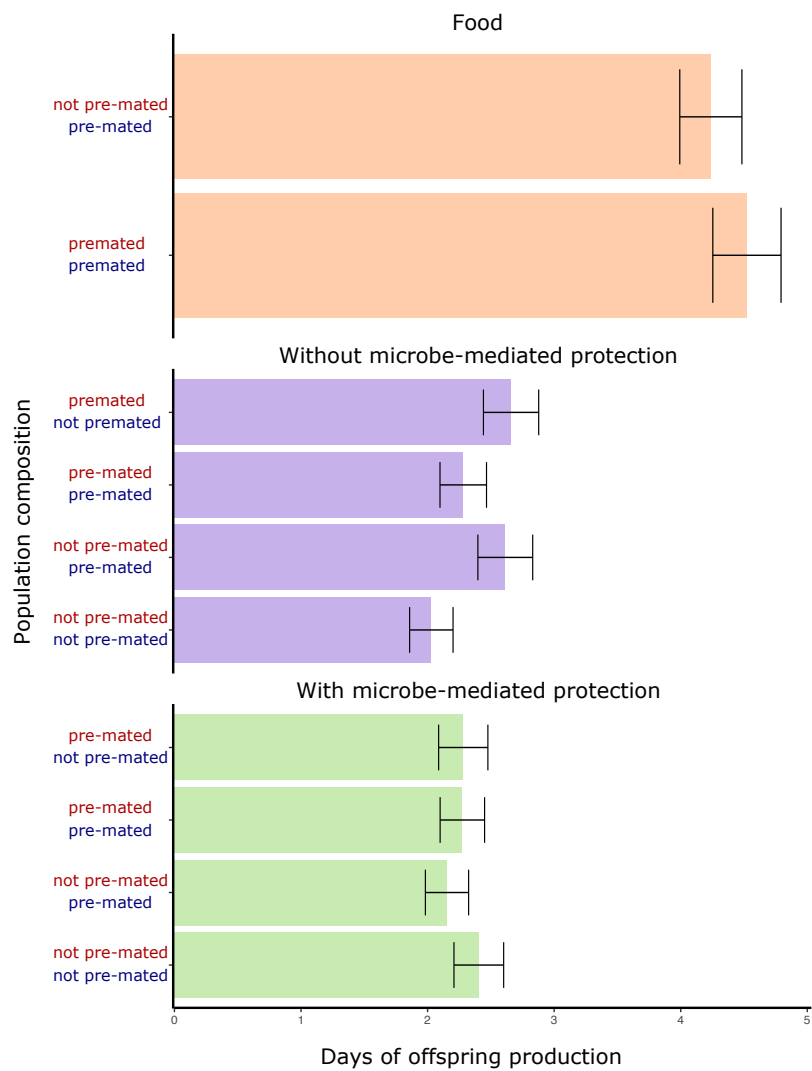

Supplement: Supplementary Figure 4 — Over a lifetime no difference between pre-mated and not pre-mated females for the days of offspring production can be observed independent on whether worms were raised on food (in orange), infected with the pathogen in the absence (in purple) or presence (in green) of MMP. (A, B) Each point represents the mean ± the standard error of the mean of three or four technical replicates. [file Image_4.pdf]

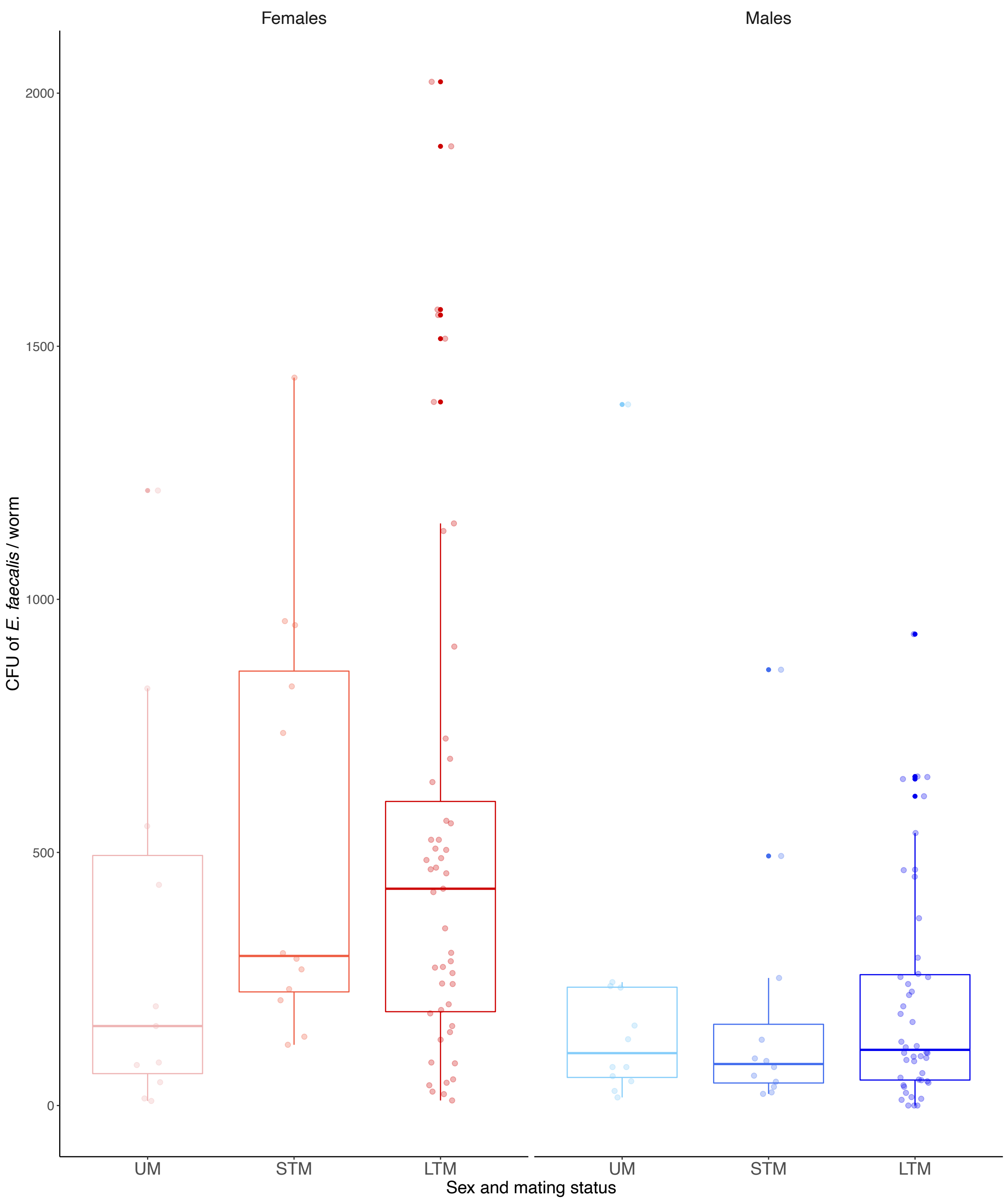

Supplement: Supplementary Figure 5 — With MMP, females are colonized by more E. faecalis than their male counter parts. After 24h infection with S. aureus, worms were filter tip washed as described before (Kloock et al., 2020) to remove all externally attached bacteria. Consequently, worms were crushed open, and the gut content was plated on E. faecalis selective medium (TSA + rifampicin), to only count E. faecalis colonies. The difference between the two sexes is significant with a linear model (LM) (LM, Sum Sq = 24.552, Df = 1, F-value = 17.6003, p = 4.99x10^-5^), while neither the mating status nor an interaction of the two show significant differences (both p>0.05). [file Image_5.pdf]
