# Supplementary material for: Sex Matters: Effects of Sex and Mating in the Presence and Absence of a Protective Microbe
Source: Front Cell Infect Microbiol. 2021 Oct 7;11:713387. doi: 10.3389/fcimb.2021.713387 (PMC8529166; doi:10.3389/fcimb.2021.713387)
Supplement: Supplementary Figure 2 — Livelong proportion of missing worms on food (A), with microbe-mediated protection (B) and without of microbe-mediated protection (C). (A) Females showed lower proportion of missing worms than males on food alone (in white). (B) Proportion of missing worms after pathogen infection (in purple) without MMP over lifetime with no differences detected. (C) Proportion of missing worms with MMP (in green) over lifetime, where males have a higher proportion of missing worms than females do. (A–C) Each point represents the mean ± the standard error of the mean of four or five biological replicates and three or four technical replicates with 20(A) or 10 worms (B, C) on each plate. [file Image_2.pdf]

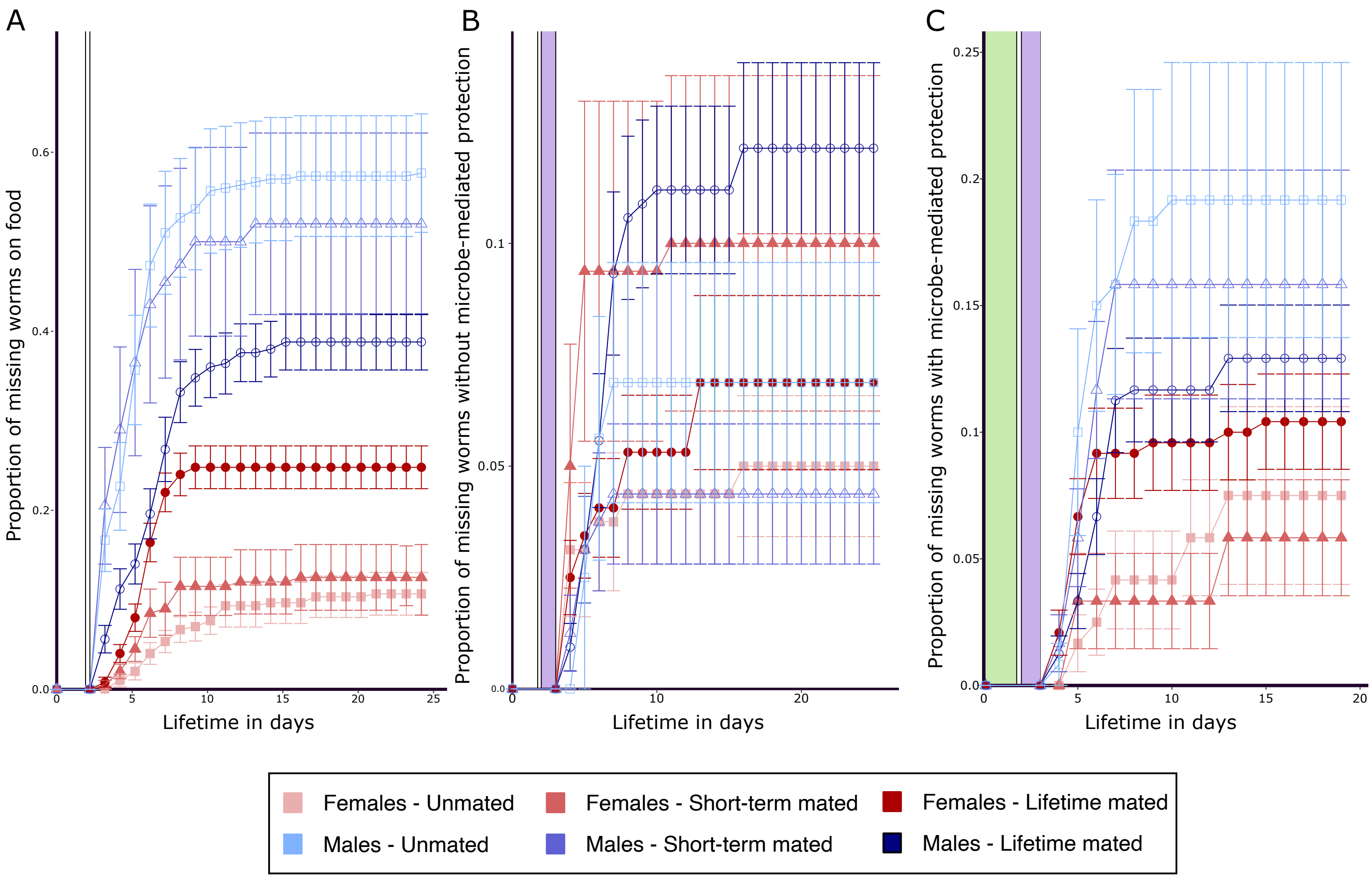
A
Proportion of missing worms on food
Lifetime in days
B
Proportion of missing worms without microbe-mediated protection
Lifetime in days
C
Proportion of missing worms with microbe-mediated protection
Lifetime in days
Females - Unmated
Females - Short-term mated
Females - Lifetime mated
Males - Unmated
Males - Short-term mated
Males - Lifetime mated
