# Supplementary material for: Sex Matters: Effects of Sex and Mating in the Presence and Absence of a Protective Microbe
Source: Front Cell Infect Microbiol. 2021 Oct 7;11:713387. doi: 10.3389/fcimb.2021.713387 (PMC8529166; doi:10.3389/fcimb.2021.713387)
Supplement: Supplementary Table 1 — Mating Treatments: Three different mating treatments (Unmated, Short-term mated and Lifetime mated) were set up for both sexes (Female in Red and Male in Blue), which were either single sex plates for each sex (Females only or Males only) or a 50:50 mixed population. Worms were left on these mating plates for 6-8h, before three different mating treatments were set up (unmated, short-term mated, and lifetime mated, the darker the colour the longer the mating period) for each sex. [file Table_1.pdf]

Females				Males			
Treatment	L1-L4	6-8h	Adult	Adult	6-8h	L1-L4	Treatment
unmated Female	Mixed Population	Females only	not premated Female				
short term mated Female	Mixed Population	50:50	premated Female				
lifetime mated Female	Mixed Population	Females only	not premated Female	not premated Male	Males only	Mixed Population	lifetime mated Male
lifetime mated Female	Mixed Population	Females only	not premated Female	premated Male	50:50	Mixed Population	lifetime mated Male
lifetime mated Female	Mixed Population	50:50	premated Female	not premated Male	Males only	Mixed Population	lifetime mated Male
lifetime mated Female	Mixed Population	50:50	premated Female	premated Male	50:50	Mixed Population	lifetime mated Male
				premated Male	50:50	Mixed Population	short term mated Male
				not premated Male	Males only	Mixed Population	unmated Male
