# Supplementary material for: Sex Matters: Effects of Sex and Mating in the Presence and Absence of a Protective Microbe
Source: Front Cell Infect Microbiol. 2021 Oct 7;11:713387. doi: 10.3389/fcimb.2021.713387 (PMC8529166; doi:10.3389/fcimb.2021.713387)
Supplement: Supplementary Table 2 — Summary of all statistical results. The following abbreviations were used: U, unmated; STM, short-term mated; LTM, Lifetime mated. [file Table_2.pdf]

Table S2: Summary of all statistical results. The following abbreviations were used: U=unmated, STM= short-term mated, LTM= Lifetime mated

Figure	Result
1A	**<u>Pathogen Infection without MMP</u>**

Technical replicates: 4

Biological replicates: 4

Number of worms: 50

Generalized linear model: glm(cbind(alive,dead) ~ Mating * Sex, family = ”Binomial”)

Effect	X^2	Df	p-value
**Mating**	108.757	2	$<2.2x10^{-16}$
**Sex**	172.383	1	$<2.2x10^{-16}$
**Interaction**	42.849	2	$4.96x10^{-10}$

Analysis of the interaction:

Generalized mixed effects model: glm(cbind(alive,dead) ~ Interaction, family = ”Binomial”)

X^2=321.01, Df=5, $p<2.2x10^{-16}$

Results of the Tukey Post-Hoc Test:

Comparison				Estimate	Std. Error	z-value	p-value
F	STM	F	UM	-0.2744	0.2189	-1.2535	0.7983
F	LTM	F	UM	-1.5667	0.1747	-8.9675	0.0000
M	UM	F	UM	0.2834	0.2474	1.1456	0.8527
M	STM	F	UM	0.1912	0.2432	0.7862	0.9673
M	LTM	F	UM	0.2821	0.2100	1.3432	0.7471
F	LTM	F	STM	-1.2923	0.1600	-8.0769	0.0000
M	UM	F	STM	0.5578	0.2372	2.3515	0.1638
M	STM	F	STM	0.4656	0.2329	1.9996	0.3269
M	LTM	F	STM	0.5565	0.1979	2.8115	0.0519
M	UM	F	LTM	1.8501	0.1971	9.3848	0.0000

M	STM	F	LTM	1.7579	0.1919	9.1613	0.0000
M	LTM	F	LTM	1.8488	0.1476	12.5289	0.0000
M	STM	M	UM	-0.0922	0.2598	-0.3548	0.9992
M	LTM	M	UM	-0.0013	0.2290	-0.0056	1.0000
M	LTM	M	STM	0.0909	0.2245	0.4048	0.9985

1B

Pathogen Infection with MMP

Technical replicates: 3

Biological replicates: 4

Number of worms: 50

Generalized mixed effects model: glm(cbind(alive, dead) ~ Mating * Sex, family = ”Binomial”)

Effect	X^2	Df	p-value
**Mating**	27.152	2	$1.271x10^{-6}$
**Sex**	34.382	1	$4.52x10^{-9}$
**Interaction**	18.416	2	0.0001002

Analysis of the interaction:

Generalized mixed effects model: glm(cbind(alive,dead) ~ Interaction, family = ”Binomial”)

X^2=80.978, Df=5, p=$5.239x10^{-16}$

Results of the Tukey Post-Hoc Test:

Comparison				Estimate	Std. Error	z-value	p-value
F	STM	F	LTM	0.8044	0.2887	2.7862	0.0546
F	UM	F	LTM	2.4503	0.5198	4.7141	0.0000
M	LTM	F	LTM	2.0833	0.3802	5.4801	0.0000
M	STM	F	LTM	1.7579	0.4331	4.0586	0.0007
M	UM	F	LTM	1.6911	0.3622	4.6695	0.0000
F	UM	F	STM	1.6459	0.5637	2.9196	0.0374
M	LTM	F	STM	1.2789	0.4384	2.9175	0.0375
M	STM	F	STM	0.9535	0.4850	1.9660	0.3410

M	UM	F	STM	0.8867	0.4228	2.0970	0.2701
M	LTM	F	UM	-0.3670	0.6156	-0.5961	0.9902
M	STM	F	UM	-0.6924	0.6497	-1.0658	0.8849
M	UM	F	UM	-0.7592	0.6047	-1.2555	0.7936
M	STM	M	LTM	-0.3254	0.5444	-0.5977	0.9901
M	UM	M	LTM	-0.3922	0.4899	-0.8007	0.9638
M	UM	M	STM	-0.0668	0.5320	-0.1256	1.0000

1C

Lifetime Analysis on Food

Technical replicates: 3

Biological replicates: 5

Number of worms: 20

Kaplan Meier Estimates

Comparisons		p-value	FDR corrected	
Female	Male	0.6199	0.6199	
U	STM	0.05122	0.0683	
U	LTM	3.33×10^{-16}	6.66×10^{-16}	***
STM	LTM	0	0	***

Comparisons		p-value	FDR-corrected	
F- U	F-STM	0.0531	0.0638	
F-U	F-LTM	0	0	***
F-STM	F-LTM	0	0	***
M-STM	M-LTM	0.0046	0.0092	***
M-LTM	M-U	0.0236	0.0354	***
M-STM	M-U	0.5932	0.5932	

Treatment	Mean	SE	Median
F-U	11.3975	0.2775	10.2
F-STM	12.3347	0.3876	10.2
F-LTM	8.1275	0.2219	8.2
M-U	10.7917	0.3158	10.2
M-STM	11.0175	0.3181	10.2
M-LTM	9.9863	0.1991	9.2

1D

<u>Lifetime Analysis after pathogen infection without MMP</u>

Technical replicates: 4

Biological replicates: 4

Number of worms: 10

Kaplan Meier Estimates

Comparisons		p-value	FDR corrected	
Female	Male	0	0	***
U	STM	0.2263	0.2633	
U	LTM	1.55×10^{-10}	3.11×10^{-10}	***
STM	LTM	4.87×10^{-7}	6.5×10^{-7}	***

Comparisons		p-value	FDR-corrected	
F- U	F-STM	0.0016	0.0032	***
F-U	F-LTM	0	0	***
F-STM	F-LTM	1.03×10^{-9}	3.09×10^{-9}	***

M-U	M-STM	0.26	0.3119	
M-U	M-LTM	0.4268	0.4268	
M-STM	M-LTM	0.0750	0.1125	

Treatment	Mean	SE	Median
F-U	8.9967	0.2415	8.0
F-STM	7.9394	0.2378	7.0
F-LTM	6.3930	0.1901	5.0
M-U	9.1519	0.2062	9.0
M-STM	9.5113	0.2147	9.0
M-LTM	8.9844	0.1842	8.0

1E

Lifetime Analysis after pathogen infection with MMP

Technical replicates: 3

Biological replicates: 4

Number of worms: 10

Kaplan Meier Estimates

Comparisons		p-value	FDR-corrected	
F- U	F-STM	0.4029	0.5996	
F-U	F-LTM	1.57×10^{-13}	4.71×10^{-13}	***
F-STM	F-LTM	0	0	***
M-U	M-STM	0.4734	0.5996	
M-U	M-LTM	0.4997	0.5996	
M-STM	M-LTM	0.7244	0.7224	

Treatment	Mean	SE	Median
F-U	8.3071	0.2532	8.0
F-STM	8.2706	0.2308	8.0
F-LTM	5.3523	0.1083	5.0

**M-U**	7.5926	0.1788	7.0
**M-STM**	7.8127	0.2009	7.0
**M-LTM**	7.3267	0.1450	7.0

2B

Activity analysis without MMP

Technical replicates: 4

Biological replicates: 4

Number of worms: 50

Generalized mixed effects model: glm(cbind(Worms at the edge, Worms not at the edge) ~ Mating * Sex, family = ”Binomial”)

Effect	X^2	Df	p-value
**Mating**	186.530	2	$<2x10^{-16}$
**Sex**	0.965	1	0.326012
**Interaction**	10.477	2	0.005307

Analysis of the interaction: glm(cbind(Worms at the edge, Worms not at the edge) ~ Interaction, family = ”Binomial”)

Generalized mixed effects model

X^2=197.14, Df=5, p<$2.2x10^{-16}$

Results of the Tukey Post-Hoc Test:

Comparison				Estimate	Std. Error	z-value	p-value
F	STM	F	LTM	-1.6716	0.3094	-5.4025	0.0000
F	UM	F	LTM	-1.5696	0.2894	-5.4238	0.0000
M	LTM	F	LTM	0.0281	0.1403	0.2006	0.9999
M	STM	F	LTM	-3.4905	0.7154	-4.8793	0.0000
M	UM	F	LTM	-2.4213	0.4231	-5.7233	0.0000
F	UM	F	STM	0.1019	0.3972	0.2567	0.9998
M	LTM	F	STM	1.6997	0.3061	5.5536	0.0000
M	STM	F	STM	-1.819	0.7653	-2.3766	0.1368
M	UM	F	STM	-0.7497	0.503	-1.4906	0.6257
M	LTM	F	UM	1.5978	0.2858	5.5903	0.0000

M	STM	F	UM	-1.9209	0.7575	-2.5359	0.0933
M	UM	F	UM	-0.8516	0.4909	-1.7349	0.4595
M	STM	M	LTM	-3.5187	0.7139	-4.9286	0.0000
M	UM	M	LTM	-2.4494	0.4206	-5.8235	0.0000
M	UM	M	STM	1.0692	0.8179	1.3073	0.7453

2C

Activity analysis with MMP

Technical replicates: 3

Biological replicates: 4

Number of worms: 50

Generalized mixed effects model: glm(cbind(Worms at the edge, Worms not at the edge) ~ Mating * Sex, family = ”Binomial”)

Effect	X^2	Df	p-value
Mating	47.812	2	$4.147x10^{-11}$
Sex	2.506	1	0.1134
Interaction	2.139	2	0.3431

Results of the Tukey Post-Hoc Test for the effect of Mating:

Comparisons		Estimate	Std. Error	z-value	p-value
STM	U	0.5704	0.1954	2.9191	0.0100
LTM	U	0.7272	0.1685	4.3172	0.0000
LTM	STM	0.1568	0.1768	0.8873	0.6472

2D

Proportion of missing worms during pathogen infection without MMP

Technical replicates: 4

Biological replicates: 4

Number of worms: 50

Generalized mixed effects model: glm(cbind(Missing Worms, Worms present on the plate) ~ Mating * Sex, family = ”Binomial”)

Effect	X^2	Df	p-value
Mating	6.4367	2	0.04002
Sex	18.4340	1	$1.759 x10^{-5}$

**Interaction**	3.1978	2	0.20212

Comparison		Estimate	Std. Error	z-value	p-value
STM	UM	-0.2841	0.1968	-1.4434	0.3152
LTM	UM	0.2023	0.1534	1.3193	0.3807
LTM	STM	0.4864	0.1710	2.8441	0.0121

2E

<u>Proportion of missing worms during pathogen infection with MMP</u>

Technical replicates: 3

Biological replicates: 4

Number of worms: 50

Generalized mixed effects model: glm(cbind(Missing Worms, Worms present on the plate) ~ Mating * Sex ,family = ”Binomial”)

Effect	X^2	Df	p-value
**Mating**	17.185	2	0.0001855
**Sex**	37.903	1	7.435×10^{-9}
**Interaction**	12.672	2	0.0014809

Analysis of the interaction:

Generalized mixed effects model: glm(cbind(Missing Worms, Worms present on the plate) ~ Interaction, family = ”Binomial”)

X^2=67.874, Df=5, p=2.836×10^{-13}

Results of the Tukey Post-Hoc Test:

Comparison				Estimate	Std. Error	z-value	p-value
F	STM	F	LTM	-0.3399	0.1594	-2.1324	0.2606
F	UM	F	LTM	-0.9478	0.1936	-4.8953	0.0000
M	LTM	F	LTM	0.2697	0.1145	2.3563	0.1646
M	STM	F	LTM	0.3249	0.1351	2.4050	0.1474
M	UM	F	LTM	0.1255	0.1409	0.8907	0.9462
F	UM	F	STM	-0.6079	0.2204	-2.7582	0.0613

	M	LTM	F	STM	0.6096	0.1556	3.9187	0.0012	
	M	STM	F	STM	0.6647	0.1713	3.8807	0.0014	
	M	UM	F	STM	0.4653	0.1759	2.6455	0.0822	
	M	LTM	F	UM	1.2175	0.1905	6.3919	0.0000	
	M	STM	F	UM	1.2726	0.2035	6.2530	0.0000	
	M	UM	F	UM	1.0733	0.2074	5.1744	0.0000	
	M	STM	M	LTM	0.0551	0.1305	0.4221	0.9982	
	M	UM	M	LTM	-0.1443	0.1365	-1.0567	0.8936	
	M	UM	M	STM	-0.1994	0.1542	-1.2929	0.7808	

2F

Lifetime analysis of proportion of missing worms on food

Technical replicates: 3

Biological replicates: 5

Number of worms: 20

Generalized mixed effects model: glm(cbind(Missing Worms, Worms present on the plate) ~ Sex * Mating, family = ”Binomial”)

Effect	X^2	Df	p-value
**Mating**	1.489	2	0.4749
**Sex**	195.959	1	$<2.2\times10^{-16}$
**Interaction**	40.583	2	1.54×10^{-9}

Analysis of the interaction:

Generalized mixed effects model: glm(cbind(Missing Worms, Worms present on the plate) ~ Interaction, family = ”Binomial”)

X^2=237.62, Df=5, p=$<2.2\times10^{-16}$

Results of the Tukey Post-Hoc Test:

Comparison				Estimate	Std. Error	z-value	p-value
F	STM	F	LTM	-0.3399	0.1594	-2.1324	0.2606
F	UM	F	LTM	-0.9478	0.1936	-4.8953	0.0000
M	LTM	F	LTM	0.2697	0.1145	2.3563	0.1646
M	STM	F	LTM	0.3249	0.1351	2.4050	0.1474

	M	UM	F	LTM	0.1255	0.1409	0.8907	0.9462
	F	UM	F	STM	-0.6079	0.2204	-2.7582	0.0613
	M	LTM	F	STM	0.6096	0.1556	3.9187	0.0012
	M	STM	F	STM	0.6647	0.1713	3.8807	0.0014
	M	UM	F	STM	0.4653	0.1759	2.6455	0.0822
	M	LTM	F	UM	1.2175	0.1905	6.3919	0.0000
	M	STM	F	UM	1.2726	0.2035	6.2530	0.0000
	M	UM	F	UM	1.0733	0.2074	5.1744	0.0000
	M	STM	M	LTM	0.0551	0.1305	0.4221	0.9982
	M	UM	M	LTM	-0.1443	0.1365	-1.0567	0.8936
	M	UM	M	STM	-0.1994	0.1542	-1.2929	0.7808

2G

Lifetime analysis of proportion of missing worms without MMP

Technical replicates: 4

Biological replicates: 4

Number of worms: 10

Generalized mixed effects model: glm(cbind(Missing Worms, Worms present on the plate) ~ Sex * Mating, family = ”Binomial”)

Effect	X^2	Df	p-value
**Mating**	2.6689	2	0.26330
**Sex**	2.6875	1	0.10114
**Interaction**	10.9124	2	0.00427

Analysis of the interaction:

Generalized mixed effects model: glm(cbind(Missing Worms, Worms present on the plate) ~ Interaction, family = ”Binomial”)

X^2=16.288, Df=5, p=0.006067

Results of the Tukey Post-Hoc Test:

Comparison				Estimate	Std. Error	z-value	p-value
F	STM	F	LTM	0.6597	0.3627	1.8188	0.4416
F	UM	F	LTM	-0.0806	0.4402	-0.1830	1.0000

	M	LTM	F	LTM	0.8855	0.3023	2.9296	0.0378	
	M	STM	F	LTM	-0.2206	0.4600	-0.4797	0.9967	
	M	UM	F	LTM	0.2578	0.3997	0.6450	0.9868	
	F	UM	F	STM	-0.7403	0.4483	-1.6513	0.5525	
	M	LTM	F	STM	0.2258	0.3140	0.7192	0.9785	
	M	STM	F	STM	-0.8804	0.4677	-1.8822	0.4016	
	M	UM	F	STM	-0.4019	0.4087	-0.9834	0.9196	
	M	LTM	F	UM	0.9661	0.4010	2.4095	0.1463	
	M	STM	F	UM	-0.1401	0.5301	-0.2643	0.9998	
	M	UM	F	UM	0.3384	0.4787	0.7068	0.9801	
	M	STM	M	LTM	-1.1062	0.4226	-2.6176	0.0885	
	M	UM	M	LTM	-0.6277	0.3561	-1.7627	0.4782	
	M	UM	M	STM	0.4785	0.4970	0.9627	0.9261	

2H

<u>Lifetime analysis of proportion of missing worms with MMP</u>

Technical replicates: 3

Biological replicates: 4

Number of worms: 10

Generalized mixed effects model: glm(cbind(Missing Worms, Worms present on the plate) ~ Sex * Mating, family = ”Binomial”)

Effect	X^2	Df	p-value
**Mating**	8.215	2	0.01645
**Sex**	160.466	1	$<2.2x10^{-16}$
**Interaction**	96.111	2	$<2.2x10^{-16}$

Analysis of the interaction:

Generalized mixed effects model: glm(cbind(Missing Worms, Worms present on the plate) ~ Interaction, family = ”Binomial”)

X^2= 264.71, Df=5, $p=<2.2x10^{-16}$

Results of the Tukey Post-Hoc Test:

Comparison				Estimate	Std. Error	z-value	p-value
F	STM	F	LTM	-0.8247	0.1263	-6.5304	0.0000
F	UM	F	LTM	-0.5836	0.1158	-5.0394	0.0000
M	LTM	F	LTM	0.1706	0.0762	2.2391	0.2095
M	STM	F	LTM	0.4786	0.0858	5.5784	0.0000
M	UM	F	LTM	0.6912	0.0822	8.4069	0.0000
F	UM	F	STM	0.2411	0.1522	1.5843	0.5956
M	LTM	F	STM	0.9953	0.1247	7.9827	0.0000
M	STM	F	STM	1.3033	0.1308	9.9659	0.0000
M	UM	F	STM	1.5159	0.1285	11.8006	0.0000
M	LTM	F	UM	0.7542	0.1141	6.6126	0.0000
M	STM	F	UM	1.0622	0.1207	8.8013	0.0000
M	UM	F	UM	1.2748	0.1182	10.7875	0.0000
M	STM	M	LTM	0.3080	0.0834	3.6928	0.0028
M	UM	M	LTM	0.5206	0.0797	6.5301	0.0000
M	UM	M	STM	0.2126	0.0889	2.3902	0.1510

5A

Wilcox-Rank-Test: wilcox.test(Plates with not pre-mated females, Plates with pre-mated females)

Technical replicates: 4

Number of plates: 4

Not pre-mated females vs. pre-mated females: p=0.004201

5B

Wilcox-Rank-Test: wilcox.test(Plates with not pre-mated females, Plates with pre-mated females)

Technical replicates: 3

Number of plates: 4

	Not pre-mated females vs. pre-mated females: p=1
**S4**	<u>Food:</u><br>Technical replicates: 3<br>Number of plates: 5<br>Wilcox-Rank-Test: wilcox.test(Plates with not pre-mated females, Plates with pre-mated females)<br>Not pre-mated females vs. pre-mated females: p=0.5222<br><br><u>Without microbe-mediated protection</u><br>Technical replicates: 4<br>Number of plates: 4<br>Wilcox-Rank-Test: wilcox.test(Plates with not pre-mated females, Plates with pre-mated females)<br>Not pre-mated females vs. pre-mated females: p=0.5227<br><u>With microbe-mediated protection</u><br>Technical replicates: 3<br>Number of plates: 4<br>Wilcox-Rank-Test: wilcox.test(Plates with not pre-mated females, Plates with pre-mated females)<br>Not pre-mated females vs. pre-mated females: p=0.9901

3
